# Die Diphtherie

**Published:** 1884-09

**Authors:** 


### BOOK REVIEWS.

---

DIE DIPHTHERIE. *Ihre Entstehung, Verhüting Und Behandlung. Von Dr. Med.* C. G. ROTHE, *Altenburg. 12 mo., pp. viii., 92. Leipzig, Ambr. Abel, 1884.*

Dr. Rothe has been favorably known for a long time as a medical writer, journalist and translator. In this little book, the principal facts in connection with the ætiology, prophylaxis, and treatment of diphtheria are collected and woven into a monograph of a remarkable character. While concise, the author is never obscure or confused in the expression of ideas. We note with pleasure the complimentary allusions to the investigations of Professor H. C. Wood and Dr. H. F. Formad, of the Med. Dept., Univ. of Pa. We heartily commend Dr. Rothe's little work to the reading public. W. W. J.

